# The Role of MreB, MreC and MreD in the Morphology of the Diazotrophic Filament of *Anabaena* sp. PCC 7120

**DOI:** 10.3390/life12091437

**Published:** 2022-09-15

**Authors:** Cristina Velázquez-Suárez, Ignacio Luque, Antonia Herrero

**Affiliations:** Instituto de Bioquímica Vegetal y Fotosíntesis, CSIC and Universidad de Sevilla, 41092 Seville, Spain

**Keywords:** heterocyst differentiation, heterocyst neck, intercellular communication, Mre proteins, peripheral peptidoglycan growth, polar peptidoglycan growth

## Abstract

The cyanobacterium *Anabaena* sp. PCC 7120 forms filaments of communicating cells. Under conditions of nitrogen scarcity, some cells differentiate into heterocysts, allowing the oxygen-sensitive N_2_-reduction system to be expressed and operated in oxic environments. The key to diazotrophic growth is the exchange of molecules with nutritional and signaling functions between the two types of cells of the filament. During heterocyst differentiation, the peptidoglycan sacculus grows to allow cell enlargement, and the intercellular septa are rebuilt to narrow the contact surface with neighboring cells and to hold specific transport systems, including the septal junction complexes for intercellular molecular transfer, which traverse the periplasm between heterocysts and neighboring vegetative cells through peptidoglycan nanopores. Here we have followed the spatiotemporal pattern of peptidoglycan incorporation during heterocyst differentiation by Van-FL labeling and the localization and role of proteins MreB, MreC and MreD. We observed strong transitory incorporation of peptidoglycan in the periphery and septa of proheterocysts and a maintained focal activity in the center of mature septa. During differentiation, MreB, MreC and MreD localized throughout the cell periphery and at the cell poles. In *mreB*, *mreC* or *mreD* mutants, instances of strongly increased peripheral and septal peptidoglycan incorporation were detected, as were also heterocysts with aberrant polar morphology, even producing filament breakage, frequently lacking the septal protein SepJ. These results suggest a role of Mre proteins in the regulation of peptidoglycan growth and the formation of the heterocyst neck during differentiation, as well as in the maintenance of polar structures for intercellular communication in the mature heterocyst. Finally, as previously observed in filaments growing with combined nitrogen, in the vegetative cells of diazotrophic filaments, the lack of MreB, MreC or MreD led to altered localization of septal peptidoglycan-growth bands reproducing an altered localization of FtsZ and ZipN rings during cell division.

## 1. Introduction

Filamentous heterocyst-forming cyanobacteria, such as the model strain *Anabaena* sp. PCC 7120 (hereafter *Anabaena*) represents genuine pluricellular bacteria in which the unit for selection has shifted from the single cell to the filament [1,2]. The *Anabaena* filament is composed of hundreds of cells, each of which is surrounded by an individual cytoplasmic membrane, whereas the outer membrane is continuous along the filament, defining a commonly shared periplasm. Indeed, the continuous periplasm has been considered a path that allows intercellular molecular exchange through the filament [3]. In addition, neighboring cells are communicated through *septal junction* complexes composed of polytopic cytoplasmic-membrane anchored proteins [4,5] that traverse the septal peptidoglycan (PG) by perforations termed nanopores, which are drilled by PG amidases [6,7,8].

Regarding the PG sacculus, the continuous filament periplasm contains a single molecule that completely encircles each cell and is engrossed in the septal location between contiguous cells [9]. In relation to its multicellular structure, *Anabaena* presents distinct characteristics in the pattern of PG incorporation. During cell division, septal incorporation occurs in the divisome to build the new septa of the resulting daughter cells. In addition, weak peripheral PG growth elongates the cells during cell growth, and continuous PG incorporation occurs in the intercellular locations along the filament, which persists after the division has been completed [10,11,12]. In model unicellular rod-shaped bacteria, the proteins MreB, MreC and MreD are involved in the spatial localization and regulation of the enzymatic machinery for lateral PG growth organized in the multiprotein complex termed the elongasome. MreB is an actin structural homolog that forms dynamic polymers associated with the inner face of the cytoplasmic membrane. MreC is a cytoplasmic membrane-anchored protein with most of its extension residing in the periplasm, and MreD is an integral membrane protein [13]. Notably, in *Anabaena*, MreB, MreC and MreD have been localized all around the cell periphery, including the mature intercellular septa, and in the divisome through all stages of cell division, having been implicated in the regulation of peripheral, medial (divisome-associated) and septal PG incorporation [10].

Depending on environmental conditions, the *Anabaena* filament can include different cell types specialized in specific functions. Thus, heterocysts are cells specialized in the fixation of atmospheric nitrogen that differentiates at semi-regular intervals along the filament under nitrogen deprivation conditions. Heterocysts supply vegetative cells with organic nitrogen. Vegetative cells fix CO_2_ through oxygenic photosynthesis and provide the heterocysts with organic carbon to be utilized as a source of reductants and as carbon skeletons for the incorporation of the ammonium resulting from N_2_ fixation [14,15].

Because the enzymatic machinery for N_2_ reduction to ammonium is extremely O_2_ sensitive, a main issue in heterocyst performance is the maintenance of a microoxic internal milieu, to which the presence of extra glycolipid and polysaccharide envelope layers, deposited during the differentiation process, contribute (see [16]). However, the entrance of N_2_ into the heterocysts is needed. Key to regulating gas exchanges is the differentiation of the heterocyst cell poles to form the specialized structure known as the heterocyst neck, which narrows the contact space between the heterocyst and the adjacent vegetative cells and represents the main path for gas exchange [14,17]. Indeed, intercellular molecular exchange involving heterocysts is thought to take place to a good extent through the heterocyst neck. Thus, although some membrane transporters of different protein families are localized through the heterocyst periphery, some others, such as the ABC-type exporters HetC, possibly involved in the export of morphogens from the heterocyst [18,19], and DevBCD-HgdD, for heterocyst glycolipid export [20], preferentially localize at the heterocyst poles. Additionally, the pentapeptide-repeat protein HglK [21] and the septal-junction associated proteins SepJ [22] and FraCD [23] are localized to the heterocyst-vegetative cell septa. Finally, the ABC-permease protein FraE [24] and the TonB-like protein SjdR [25] have been identified as required for the constriction and arrangement of the heterocyst polar structures.

Besides peripheral PG growth required for cell enlargement, the differentiation of the heterocyst neck also appears to involve PG remodeling/turnover see [11]. Consistent with this, mutations in genes encoding enzymes involved in different steps of PG synthesis lead to impaired heterocyst differentiation [26,27,28,29]. Moreover, PG septal disk diameter, nanopore diameter and nanopore number are larger in septa between vegetative cells and heterocysts than in septa between two vegetative cells [24], and mutants deficient in the PG amidases AmiC1 [7] or AmiC3 [8] are unable of diazotrophic growth.

In this work, we have monitored the incorporation of PG and the localization of the MreB, MreC and MreD proteins in *Anabaena* during heterocyst differentiation and in mature diazotrophic filaments. We have also addressed the influence of MreB, MreC and MreD on peripheral, medial and septal PG growth, the filament geometry and the formation of heterocyst-vegetative cell connections.

## 2. Materials and Methods

### 2.1. Strains and Growth Conditions

*Anabaena* sp. strain PCC 7120 and mutant strains (strains and plasmids used in this work are listed in Table S1) were grown in BG11 medium (containing NaNO_3_ as a nitrogen source) [30] containing ferric citrate instead of ferric-ammonium citrate. Cultures were incubated at 30 °C with illumination (12 μE m^−2^ s^−1^ white light from Osram LED lamps 16.4 W/4000 K), in Erlenmeyer flasks with shaking (100 rpm) or in plates of medium solidified with 1% Difco agar. For the mutants, media were supplemented with antibiotics: spectinomycin dihydrochloride pentahydrate (Sp) and streptomycin sulfate (Sm) at 5 μg mL^−1^ each in solid medium or 2.5 μg mL^−1^ each in liquid medium (CSCV2, CSCV6, CSCV7, CSCV8, CSSC19, CSAV39), with neomycin sulfate (Nm) at 25 μg mL^−1^ in solid medium or 5 μg mL^−1^ in liquid medium (CSS89, CSCV1, CSCV4), or with Sm, Sp and Nm (CSCV14, CSCV15, CSCV16, CSCV17, CSCV18, CSCV19, CSCV20, CSCV21, CSCV22). For the experiments described in this work, BG11-grown filaments of the indicated strains were transferred to (at a cell density corresponding to 0.5 μg chlorophyll mL^−1^) and incubated in BG11_0_ medium [30] lacking NaNO_3_ and antibiotics. Chlorophyll *a* (Chl) content of the cultures was determined after extraction with methanol [31]. In *Anabaena*, 1 μg Chl corresponds to ca. 3.3 × 10^6^ cells [32].

### 2.2. Strain Constructions

Strains CSCV17, CSCV18 and CSCV19 bear a *sepJ-gfpmut2* fusion gene in a *mreB*, *mreC* or *mreD* mutant background, respectively. To generate strain CSCV19, the conjugative plasmid pCSVT22 (encoding Nm resistance and including sequences of the 3’ part of *Anabaena sepJ* fused to *gfpmut2*) [23] was transferred to strain CSCV2 (*mreD*) by conjugation, and one clone in which the plasmid was inserted by single cross-over into *sepJ* was selected and verified by PCR. To generate strains CSCV17 and CSCV18, the insert of pCSVT22 was amplified with oligonucleotides alr2338-BamH1 and gfp-BamH1 (listed in Table S1) and cloned in the conjugative vector pCSV3 encoding Sm/Sp resistance [33] generating plasmid pCSCV38, which was transferred to strains CSCV1 (*mreB*) and CSCV4 (*mreC*) by conjugation. Clones in which pCSCV38 was inserted by single cross-over into *sepJ* were selected and verified by PCR.

### 2.3. Microscopy

For Van-FL labeling and detection, filaments were treated as described [10] and visualized with a Leica DM6000B fluorescence microscope and a FITCL5 filter (excitation band-pass, 480/40; emission band-pass, 527/30), and photographed with an ORCA-ER camera (*Hamamatsu*). GFP fluorescence was monitored with an Olympus TCS SP2 confocal laser-scanning microscope equipped with an HCX PLAN-APO 63 × 1.4 NA oil immersion objective (excitation, 488-nm; collection, 500–540 nm for GFP or 630–700 nm for cyanobacterial autofluorescence) or with an Olympus FLUOVIEW FV3000 (hyper-resolution) confocal laser-scanning microscope equipped with a UPlanApo 60 × 1.5 NA oil immersion objective (excitation, 488-nm; collection 500–540 nm for GFP or excitation, 640 nm; collection, 650–750 nm for cyanobacterial autofluorescence).

## 3. Results

### 3.1. PG Growth during Heterocyst Differentiation in Anabaena

**Figure 1:**
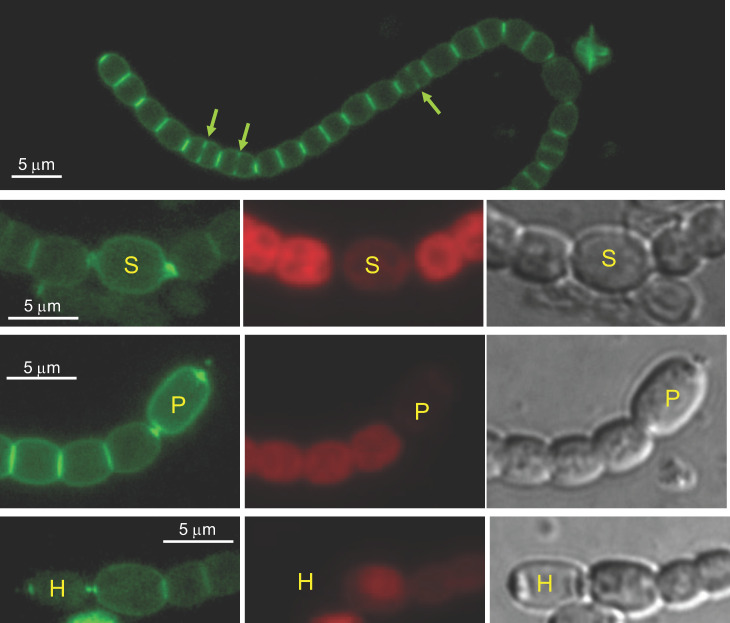
Van-FL staining of filaments of *Anabaena* sp. strain PCC 7120 during heterocyst differentiation. Filaments grown in BG11 medium were transferred to BG11_0_ (lacking combined nitrogen) medium and incubated under culture conditions. After 24 (row 2) or 48 h (rows 1, 3 and 4), samples were stained with Van-FL and observed under a fluorescence microscope and photographed. Van-FL fluorescence (green), cyanobacterial autofluorescence (red), and bright-field images are shown. Cells in early stages of differentiation (S), immature heterocysts (P) and mature heterocysts exhibiting polar refringent cyanophycin granules (H) (see the text for details) are indicated. Green arrows in row 1 point to fluorescence matching the divisome complex.

Van-FL is a fluorescent derivative of the antibiotic vancomycin that binds to the PG precursor Lipid II in the periplasm, either bound or not to the sacculus but not involved in crosslinking, thus providing a probe for nascent PG synthesis [34]. We used Van-FL to label *Anabaena* filaments during heterocyst differentiation upon transfer of filaments grown in BG11 medium (containing nitrate) to BG11_0_ medium lacking combined nitrogen. The differentiation process can be followed through a succession of cellular signals that include progressive loss of red fluorescence due to photosynthetic pigment rearrangement, cell elongation and enlargement, deposition of extracellular envelope layers, and finally, accumulation in refringent polar granules of cyanophycin resulting from N_2_ fixation. In the differentiating cells, the localization of Van-FL fluorescence appeared to follow a sequence through the different stages of the differentiation process (Figure 1). Thus, at early stages (cells already enlarged but with no conspicuous change in morphology and still detectable red fluorescence), Van-FL fluorescence was detected as a homogenous peripheral signal and in foci localized in the heterocyst neck. In the neighboring vegetative cells, fluorescence was detected at the pole contiguous to the differentiating heterocyst (see the second row in Figure 1; see also [35]). Afterward, concomitant with morphological reshaping and red-fluorescence loss, the peripheral signal in the differentiating cells appeared more pronounced, especially in the polar regions. Moreover, a strong label was detected in the pole proximal to the heterocyst of the neighboring vegetative cells (see the third row in Figure 1). Later, in mature heterocysts (showing conspicuous polar granules), the peripheral signal faded, and fluorescence was more restricted to foci in the center of the heterocyst poles and the neighboring vegetative cell pole (see the fourth row in Figure 1). These observations suggest that during heterocyst differentiation, a phase of peripheral PG growth occurs at intermediate stages of differentiation (as previously noted [11]), being this a transient event. In addition, continuous activity of PG remodeling appears to be maintained at the poles of heterocysts and neighboring vegetative cell poles.

Regarding the vegetative cells of filaments incubated in BG11_0_ medium, continuous peripheral Van-FL fluorescence and strong fluorescence bands in the intercellular regions, reminiscent of the pattern found in filaments growing with combined nitrogen [10], were observed (first row in Figure 1). As in the latter, in actively-growing diazotrophic filaments, the septal bands alternate in intensity, with the intensity detected in the recently formed septa being lower than in the older septa. Additionally, midcell fluorescence progressing inwards from the cell surface, matching the progressing septum under construction, could be detected in dividing cells.

### 3.2. Localization of MreB, MreC and MreD during Heterocyst Differentiation

**Figure 2:**
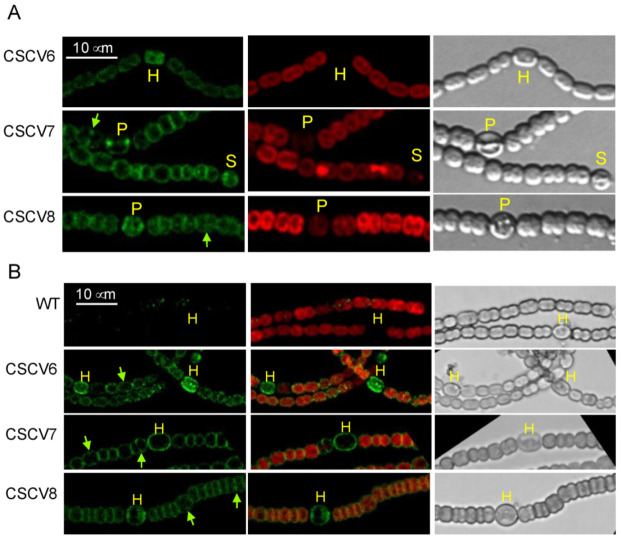
Localization of MreB, MreC and MreD in *Anabaena*. Strain PCC 7120 and their derivatives CSCV6 (sfGFP-MreB), CSCV7 (sfGFP-MreC) and CSCV8 (sf-GFP-MreD) were grown in BG11 medium, transferred to BG11_0_ medium and incubated under culture conditions. After 24 h, filaments were visualized by confocal microscopy with TCS (**A**) or FLUOVIEW (**B**) equipment. GFP fluorescence (green), cyanobacterial autofluorescence (red), merged GFP and cyanobacterial autofluorescence, and bright-field images are shown. Cells in early stages of differentiation (S), immature heterocysts (P) and mature heterocysts (H) are indicated. Green arrows point to GFP fluorescence matching divisome complexes. Magnification is the same for all micrographs in (**A**) or (**B**).

We studied the localization of MreB, MreC and MreD in filaments of *Anabaena* subjected to N-stepdown using protein fusions to GFP. For that, we used the previously generated strains CSCV6, CSCV7 and CSCV8 that express the genes *sfgfp-mreB*, *sfgfp-mreC* or *sfgfp-mreD* (encoding superfolder GFP fused to MreB, MreC or MreD, respectively) preceded by the native promoter of the *mreBCD* operon, located on an ectopic chromosomal locus, keeping an intact *mreBCD* operon in its native genomic location [10]. GFP fluorescence was monitored in filaments of CSCV6, CSCV7 and CSCV8 grown with nitrate and transferred to a medium lacking combined nitrogen. In the three strains, fluorescence was localized over the periphery of cells in the early stages of differentiation, as well as of immature and mature heterocysts (exhibiting complete loss of red fluorescence and conspicuous polar granules) (Figure 2), and tended to become more diffuse in older heterocysts (not shown). GFP fluorescence could also be detected frequently as foci in the heterocyst poles (Figure 2A; see CSCV8 in Figure 2B).

In the vegetative cells of strains CSCV6, CSCV7 and CSCV8, GFP fluorescence could also be detected in the cell periphery during active growth, and at midcell in signals progressing inwards from the periphery in the dividing cells (Figure 2). As previously noted [10], especially cells of CSCV7 and CSCV8 appeared bigger and rounder than those of the wild type. This could indicate some interference of the fusion proteins with the function of the corresponding native proteins. Nonetheless, the GFP fluorescence localization’s specificity and consistency with the signals produced by GFP-MreB (in CSCV6) support that GFP-MreC and GFP-MreD are reporting the physiological localization of MreC and MreD, respectively.

### 3.3. Influence of MreB, MreC and MreD in PG Growth during Heterocyst Differentiation

**Figure 3:**
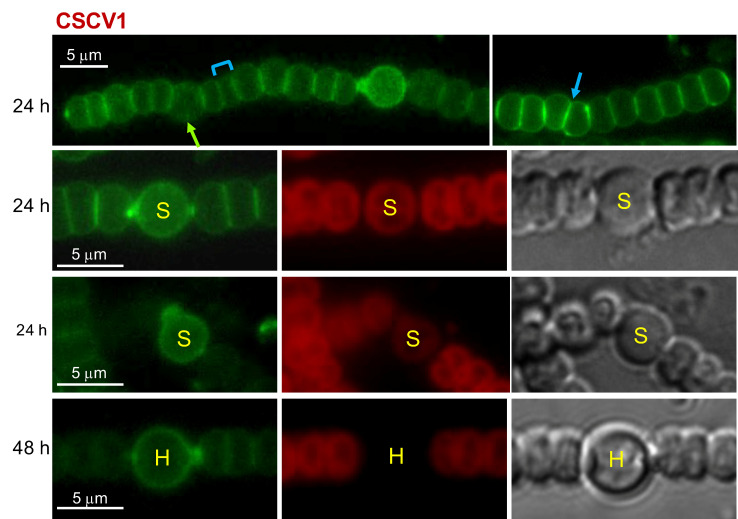
Van-FL staining of *Anabaena mreB* mutant. Strain CSCV1 (*mreB*) was treated under the same conditions described for the WT (PCC 7120) in Figure 1. Van-FL fluorescence (green), cyanobacterial autofluorescence (red) and bright-field images are shown. To improve visibility, contrast/brightness is different for the image at left in row 1. The bracket indicates a cell compartment with disparate size; the blue arrow points to a tilted fluorescent band and the green arrow to fluorescence matching the divisome. Cells in the early stages of differentiation (S) and mature heterocysts (H) are indicated.

**Figure 4:**
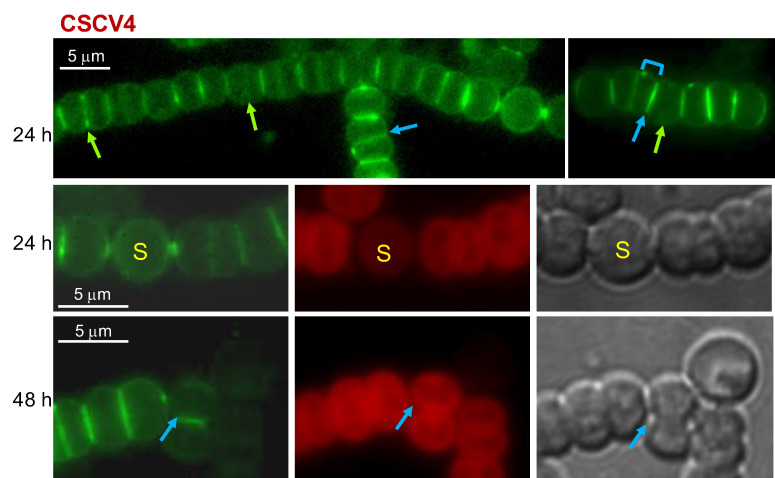
Van-FL staining of *Anabaena mreC* mutant strain CSCV4. See the legend in Figure 3 for details. To improve visibility, contrast/brightness is different for the image at left in row 1.

**Figure 5:**
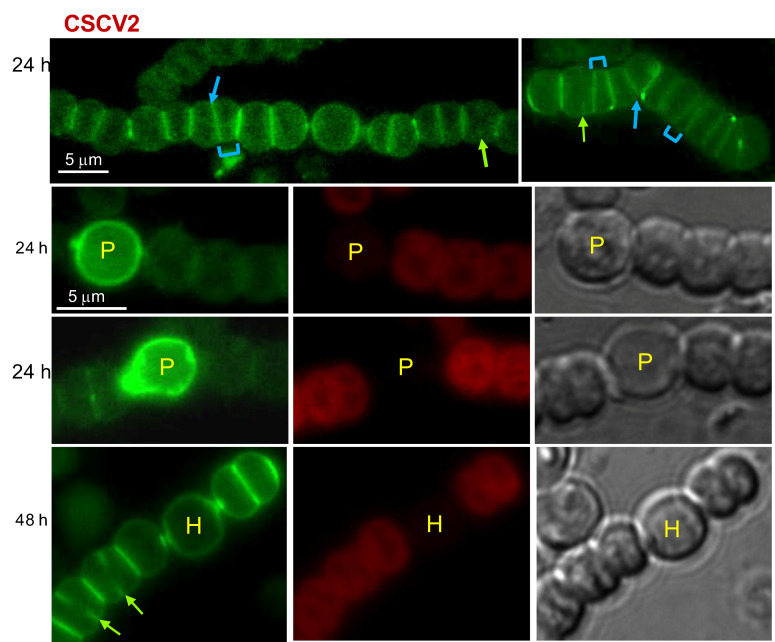
Van-FL staining of *Anabaena mreD* mutant strain CSCV2. See the legend in Figure 3 for details. Immature heterocysts (P) and mature heterocysts (H) are indicated. To improve visibility, contrast/brightness differs for the images in row 1.

Strains CSCV1, CSCV4 and CSCV2 contain only inactivated versions of *mreB*, *mreC*, or *mreD*, respectively. These mutants show strong morphological alterations, forming cells, including heterocysts, that are larger and rounder than wild-type cells [36]. Van-FL staining was performed in filaments of strains CSCV1, CSCV4 and CSCV2 grown with nitrate and incubated in the absence of combined nitrogen. Fluorescence localization was compared to the wild-type (shown in Figure 1). Regarding fluorescence distribution in differentiating cells, some heterocysts with peripheral fluorescence and septal foci could be detected in CSCV1 (see the second row in Figure 3), CSCV4 (see first and the second row in Figure 4) and CSCV2 (see the second row in Figure 5). However, instances of deformed septal fluorescence distribution or aberrantly increased labeling were also found (see the third row in Figure 3, for CSCV1, and the third row in Figure 5, for CSCV2). In the three mutants, but especially in CSCV4, few mature heterocysts with conspicuous polar granules were detected after 48 h of incubation in the absence of combined nitrogen, consistent with the previous description of retarded expression of nitrogenase activity [36]. Frequently, heterocysts with conspicuous peripheral labeling could still be detected at 48 h (see the fourth row in Figure 3 for CSCV1 and Figure 5 for CSCV2).

In vegetative cells (see the first row in Figure 3, Figure 4 and Figure 5), the three mutants showed peripheral, septal and midcell Van-FL labeling. However, the consecutive septal bands appeared frequently tilted with regard to each other or at irregular distances from each other (compare first rows in Figure 1, Figure 3, Figure 4 and Figure 5). Inspection of 100 to 120 septal bands of each PCC 7120, CSCV1, CSCV4 and CSCV2 incubated for 24 and 48 h in BG11_0_ medium showed that, in the mutants, 29–40% septal bands appeared conspicuously tilted with regard to a consecutive band. In the wild type, 95–98% appeared parallel to its consecutive band (Mann–Whitney tests indicated fully significant differences, *p* < 0.001, for any comparison between a mutant and the WT, and no significant differences between the mutants). CSCV4 even showed some instances where the division plane appeared inverted (see row 3 in Figure 4). In addition, abundant cell debris from cell lysis was observed especially in CSCV1 and CSCV4.

### 3.4. Localization of FtsZ and ZipN in Mre Mutants

Because heterocyst differentiation is linked to inhibition of cell division (see [15]) and because, as shown above, MreB, MreC and MreD are also localized to the divisome, we asked whether the Mre proteins affected the localization of FtsZ and ZipN during the differentiation process. FtsZ initiates the polymerization of the division ring (see [37]), and ZipN is the main tether for binding FtsZ to the cytoplasmic membrane and organizer of the divisome in *Anabaena* [38]. We monitored FtsZ localization using strains that express an *ftsZ-gfp* reporter from the P*_ftsZ_* promoter in the WT background (strain CSSC19 [37]) and in the *mreB* (strain CSCV20), *mreC* (strain CSCV21) and *mreD* (strain CSCV22) mutant backgrounds [10].

**Figure 6:**
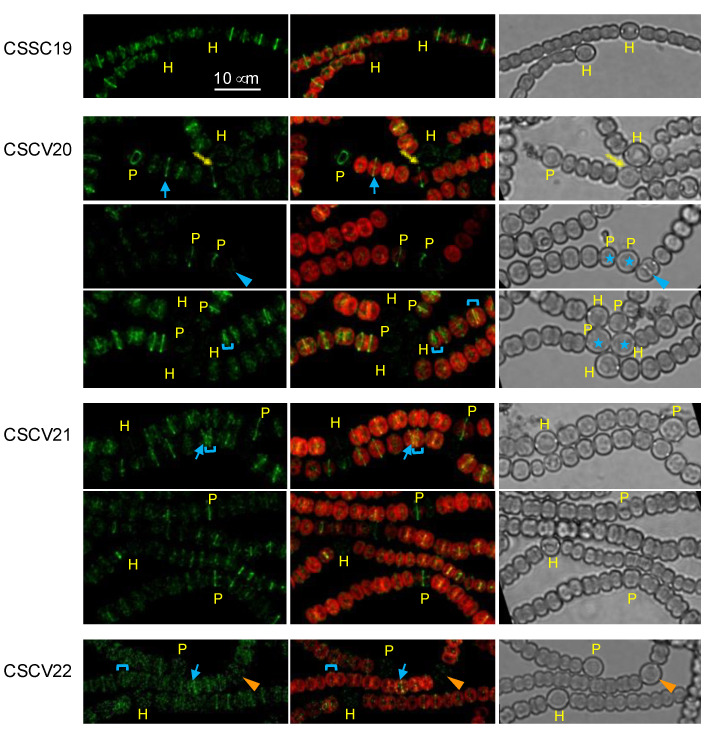
Localization of FtsZ in *mreB*, *mreC* and *mreD* mutants. Strains CSSC19 (*ftsZ*-*gfpmut2* in WT background), CSCV20 (*ftsZ*-*gfpmut2* in CSCV1 background), CSCV21 (*ftsZ*-*gfpmut2* in CSCV4 background) and CSCV22 (*ftsZ*-*gfpmut2* in CSCV2 background) were treated as above described for Figure 2. After 48 h, filaments were visualized by confocal microscopy with FLUOVIEW equipment. GFP fluorescence (green), merged GFP and cyanobacterial autofluorescence (red), and bright-field images are shown. To improve visibility, contrast is higher for the green image of CSCV22. Blue arrows point to tilted fluorescence bands, and blue brackets to cell compartments with disparate sizes. Immature (P) and mature heterocysts (H) are indicated. Blue arrowheads point to an apparent instance of septation in mature heterocysts; orange arrowheads to a mature heterocyst with Z-ring fluorescence; and blue asterisks to contiguous heterocysts. Magnification is the same for all micrographs.

Upon N-stepdown, fluorescent FtsZ-rings could be detected in vegetative cells of filaments of all strains (Figure 6). However, in the mutant backgrounds, some rings were tilted compared to those of neighboring cells, and in some places, the distance between two consecutive Z-rings was irregular. In the differentiating cells described for strain CSSC19 (39), Z-rings were detected during differentiation but not in mature heterocysts. CSSC19 mostly exhibited mature heterocysts 48 h after N-stepdown, whereas in CSCV20, CSCV21 and CSCV22, cells exhibiting fluorescent rings were still abundant at that time, consistent with retardation of heterocyst differentiation. Of note is the observation of some aberrant events in CSCV20, such as the differentiation of consecutive cells (see third and fourth rows in Figure 6), which is very rare in the wild type, or the presence of a Z-ring (see the seventh row in Figure 6) or apparent septation (see the third row in Figure 6) in mature heterocysts.

The effect of the lack of MreB, MreC or MreD on the localization of ZipN was studied in strains that express an *sfgfp-zipN* reporter gene from the P*_zipN_* promoter in the WT background (strain CSAV39 [39]) and the *mreB* (strain CSCV14), *mreC* (strain CSCV15) and *mreD* (strain CSCV16) mutant backgrounds [10].

**Figure 7:**
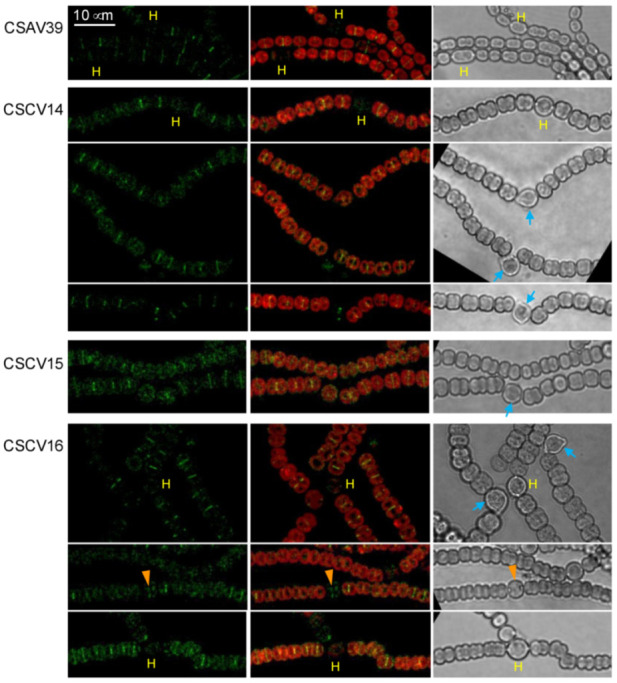
Localization of ZipN in *Anabaena mreB*, *mreC* and *mreD* mutants. Strains CSAV39 (*sfgfp-zipN* in WT background), CSCV14 (*sfgfp-zipN* in CSCV1 background), CSCV15 (*sfgfp-zipN* in CSCV4 background) and CSCV16 (*sfgfp-zipN* in CSCV2 background) were treated as described above for Figure 2, and after 24 h (48 h for rows 7 and 8), filaments were visualized by confocal microscopy with FLUOVIEW equipment. GFP fluorescence (green), merged GFP and cyanobacterial autofluorescence (red), and bright-field images are shown. Mature heterocysts (H) are indicated. Orange arrowheads point to an instance of midcell GFP-ZipN in a mature heterocyst. Blue arrows point to heterocysts deviated from the filament longitudinal plane or with aberrant polar morphology. Magnification is the same for all micrographs.

Upon N-stepdown, strain CSAV39 presented midcell fluorescent bands in vegetative cells, and this signal was progressively lost in the differentiating cells, being absent in mature heterocysts (Figure 7; [39]). In CSCV14, CSCV15 and CSCV16, the midcell GFP-ZipN fluorescence was also lost in most mature heterocysts, although in some of them, some signals could still be detected (see the heterocyst in the seventh row in Figure 7 for CSCV16). In the three mutant backgrounds, but especially in CSCV15, fluorescence appeared more dispersed than in the wild-type background (strain CSAV39). Of note is the observation that in the three mutant backgrounds some heterocysts deviated from the filament longitudinal plane. Moreover, in CSCV14 and CSCV16, heterocysts with aberrant polar morphology even provoking filament breakage were observed (Figure 7).

### 3.5. Localization of SepJ in Mre Mutants

Because the *mreB*, *mreC* and *mreD* mutants are impaired in diazotrophic growth [36], and because, as shown above, MreB, MreC and MreD are also localized to the heterocyst poles and the neighboring vegetative cell poles, we asked whether Mre proteins had any influence on the localization of the septal protein SepJ. To study that, we generated strains CSCV17, CSCV18 and CSCV19, which express a SepJ-GFP reporter from the native *sepJ* gene promoter as the only SepJ version (see [22]), in *mreB*, *mreC* or *mreD* background, respectively. Strain CSS89 expresses the same reporter in the wild-type background [38].

**Figure 8:**
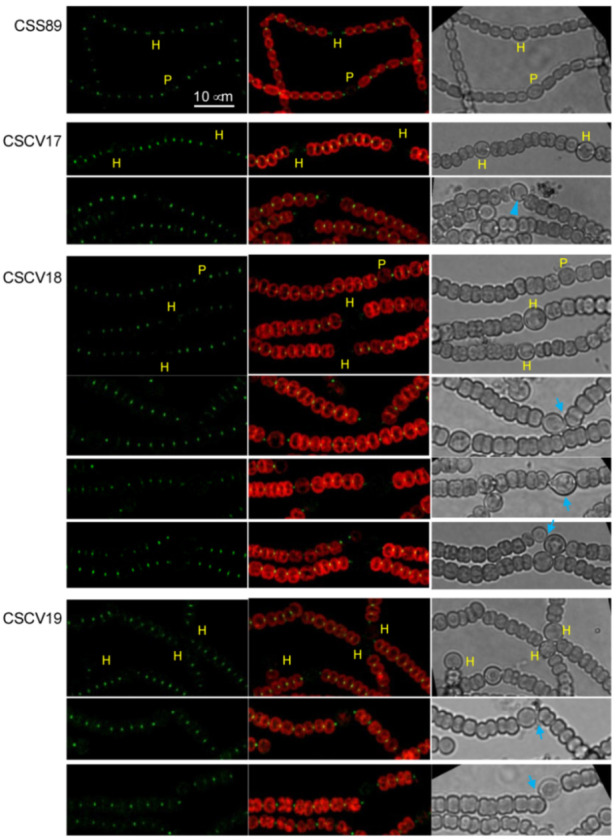
Localization of SepJ in *Anabaena mreB*, *mreC* and *mreD* mutants. Strains CSS89 (*sepJ-gfpmut2* in WT background), CSCV17 (*sepJ-gfpmut2* in CSCV1 background), CSCV18 (*sepJ-gfpmut2* in CSCV4 background) and CSCV19 (*sepJ-gfpmut2* in CSCV2 background) were treated as described above for Figure 2. After 24 h (rows 5, 9 and 10) or 48 h (rows 1–4 and 6–8), filaments were visualized by confocal microscopy with FLUOVIEW equipment. GFP fluorescence (green), merged GFP and cyanobacterial autofluorescence (red), and bright-field images are shown. Immature (P) and mature heterocysts (H) are indicated. Blue arrows point to heterocysts deviated from the filament longitudinal plane or with aberrant polar morphology. The blue arrowhead points to a heterocyst with polar granules deviated from the cell equator. Magnification is the same for all micrographs.

In strain CSS89, GFP fluorescence appeared as conspicuous focused central spots in the vegetative cell and heterocyst poles (Figure 8; see also [22]). In CSCV17, CSCV18 and CSCV19, a similar fluorescence distribution could be observed, although mature heterocysts with very weak or undetectable fluorescence were also observed (Figure 8). Notably, some heterocysts in which the cyanophycin polar granules were displaced from the cell equator were detected in strain CSCV17 (see the third row in Figure 8). Additionally, in the three mutant backgrounds, heterocysts with aberrant polar morphology were frequently observed, as well as some points of filament bending, eventually leading to filament breakage, at a heterocyst-vegetative cell connection, sometimes involving a heterocyst pole devoid of SepJ (Figure 8).

## 4. Discussion

Under conditions of nitrogen scarcity, the *Anabaena* filament develops into a diazotrophic filament that includes cells specialized for the fixation of atmospheric nitrogen called heterocysts. The differentiation of vegetative cells into heterocysts includes multiple morphological, structural and metabolic changes to permit the efficient operation of the N_2_ fixation machinery. Heterocysts are larger in size and longer in cell aspect than their mother cells, either of filaments that had grown with combined nitrogen or vegetative cells of diazotrophic filaments [36]. We have shown here conspicuous peripheral labeling with Van-FL of cells in early stages of differentiation, indicating incorporation of PG that would allow for envelope expansion during cell enlargement. Consistent with these results, fluorescent HADA, which incorporates into PG, has been found to label proheterocysts at a stage in which they were also labeled with Alcian blue, which indicates that at least the polysaccharide envelope layer had already been deposited [11]. However, the low turnover of the HADA signal may make Van-FL labeling more adequate for studying the dynamics of PG incorporation. Consequently, we have observed that the peripheral Van-FL labeling decreased in mature heterocysts, suggesting that lateral PG growth is transitory.

Previous observations support the involvement of the MreB, MreC and MreD proteins in PG incorporation during heterocyst differentiation. Thus, the expression of the *mreBCD* operon transiently increases in differentiating cells, returning to a level similar to that of vegetative cells in mature heterocysts, and even decreasing thereafter [36]. Moreover, the *mreB*, *mreC* or *mreD* mutants’ heterocysts are no longer rod-shaped. Instead, the axis parallel to the filament is shorter than that perpendicular to the filament [36]. In addition, we have shown here that MreB, MreC and MreD localize to the periphery and the cell poles of differentiating cells and heterocysts (Figure 2). Nonetheless, we have detected peripheral PG incorporation in the rounded differentiating cells of the *mreB*, *mreC* and *mreD* mutants (Figure 3, Figure 4 and Figure 5). These results indicate that whereas the Mre proteins are not strictly required for PG incorporation in the differentiating cells, they are necessary for a checked peripheral PG growth leading to cell elongation. Consistent with this notion, we have detected instances of aberrantly strong peripheral incorporation of Van-FL, at least in the case of the *mreD* mutant (Figure 5). Although we cannot rule out that this effect results from easier access of Van-FL to the periplasm due to an alteration of the outer membrane, it could also indicate deregulated PG growth in the mutants.

The polar regions of heterocysts represent differentiated structures that allow for controlled intercellular communication with the neighboring cells in the filament. Differentiation of the heterocyst neck includes polar localization of specific membrane transporters and the septal junction complexes traversing the septal PG through nanopores. In addition to peripheral growth, intense activity of PG incorporation at the poles of differentiating heterocysts was detected using HADA [11]. We have observed conspicuous Van-FL labeling in the differentiating cell poles covering the heterocyst neck at the early stages of differentiation (Figure 1), as well as the progression of the wide signals observed early into focal polar labeling, similar to those detected in the vegetative cell poles contiguous to a heterocyst (Figure 1). These observations indicate a strong PG incorporation during the formation of the heterocyst neck that leads to persistent, more focused incorporation at the mature heterocyst poles, likely serving in the formation and maintenance of septal communication arrays.

MreB, MreC and MreD are present in the heterocyst poles (Figure 2). This, together with the instances of increased or aberrant Van-FL distribution found in the poles of the heterocysts of the *mre* mutants (Figure 3, Figure 4 and Figure 5), suggest the involvement of the Mre proteins in reshaping of septal PG during heterocyst polar differentiation and maintenance. In support of this notion, we have observed that in the *mreB*, *mreC* and *mreD* mutants, heterocysts frequently lack SepJ-GFP fluorescence spots (Figure 8), which suggests an influence of the Mre proteins in the localization of septal structures. Moreover, in those mutants, heterocysts with aberrant polar morphology, including those in which polar granules were displaced from the cell equator and those in which the linearity of the filament was lost at a heterocyst-vegetative cell connection, even leading to filament breakage at that point, were found. All these polar alterations could provoke impaired intercellular exchange between heterocysts and the neighboring vegetative cells, contributing to the impaired diazotrophic growth exhibited by the *mre* mutants [36].

Finally, the pattern of Van-FL labeling in the vegetative cells of filaments in the process of differentiation and during diazotrophic growth (First row in Figure 1) appeared similar to that previously found in filaments growing with combined nitrogen, with peripheral, septal and midcell labeling (e.g., [10]). Additionally, under diazotrophic conditions, inactivation of *mreB*, *mreC* or *mreD* led to alterations in the pattern of septal bands, which were frequently disposed forming angles or at irregular distances to each other (First row in Figure 3, Figure 4 and Figure 5), alterations that reproduce those found in the disposition of the FtsZ and ZipN rings (Figure 6 and Figure 7). FtsZ and ZipN rings are dismantled in the cells differentiating into heterocysts at intermediate stages of differentiation [39,40]. This leads to a loss of cell-division capacity, considered to be a crucial step in setting the commitment to terminal differentiation ([41]; see [15]). Indeed, mutations that increase the persistence of division rings in differentiating cells have been considered to inhibit the completion of differentiation [39]. Here, we have observed that 48 h after N-stepdown, some immature heterocysts with conspicuous FtsZ rings were still present in the *mreB*, *mreC* and *mreD* mutant backgrounds but not in the wild-type background (Figure 6). Even some apparently mature heterocysts exhibiting an FtsZ ring (Figure 6) or a ZipN ring (Figure 7) were detected in the *mreD* mutant. These observations are also indicative of retardation of differentiation in the *mre* mutants. Whether, in addition to influencing the division-ring topology, MreB, MreC or MreD also have a role in the inhibition of Z-ring establishment during heterocyst differentiation is an issue worth future investigation.

## Supplementary Materials

The following supporting information can be downloaded at: https://www.mdpi.com/article/10.3390/life12091437/s1, Table S1: Cyanobacterial strains, plasmids and oligonucleotides used in this work.
