# Supplementary material for: The Role of MreB, MreC and MreD in the Morphology of the Diazotrophic Filament of Anabaena sp. PCC 7120
Source: Life (Basel). 2022 Sep 15;12(9):1437. doi: 10.3390/life12091437 (PMC9503725; doi:10.3390/life12091437)
Supplement: Supplementary file 1 [file life-12-01437-s001.zip › life-1863267-supplementary.pdf]

Table S1. Cyanobacterial strains, plasmids and oligonucleotides used in this work

**Strain**	**Genotype**	**Resistance**	**Source**
Anabaena sp. PCC 7120	WT		Pasteur Culture Collection
CSCV1	*mreB*::C.K1	Nm	36
CSCV2	*mreD*::C.S3	Sm, Sp	36
CSCV4	*mreC*::C.K1	Nm	36
CSCV6	*thrS2*::P_{mreB}-*sfgfp-mreB*	Sm, Sp	10
CSCV7	*thrS2*::P_{mreB}-*sfgfp-mreC*	Sm, Sp	10
CSCV8	*thrS2*::P_{mreB}-*sfgfp-mreD*	Sm, Sp	10
CSSC19	P_{ftsZ}-*ftsZ-gfpmut2*	Sm, Sp	37
CSCV20	P_{ftsZ}-*ftsZ-gfpmut2*, *mreB*	Nm, Sm, Sp	10
CSCV21	P_{ftsZ}-*ftsZ-gfpmut2*, *mreC*	Nm, Sm, Sp	10
CSCV22	P_{ftsZ}-*ftsZ-gfpmut2*, *mreD*	Nm, Sm, Sp	10
CSAV39	P_{zipN}-*sfgfp-zipN*	Sm, Sp	39
CSCV14	P_{zipN}-*sfgfp-zipN*, *mreB*	Nm, Sm, Sp	10
CSCV15	P_{zipN}-*sfgfp-zipN*, *mreC*	Nm, Sm, Sp	10
CSCV16	P_{zipN}-*sfgfp-zipN*, *mreD*	Nm, Sm, Sp	10
CSS89	*sepJ-gfpmut2*	Nm	38
CSCV17	*sepJ-gfpmut2*, *mreB*	Nm, Sm, Sp	This study
CSCV18	*sepJ-gfpmut2*, *mreC*	Nm, Sm, Sp	This study
CSCV19	*sepJ-gfpmut2*, *mreD*	Nm, Sm, Sp	This study

**Plasmid**	**Description**	**Resistance marker**	**Source**
pCSCV38	pCSV3 carrying *sepJ-gfpmut2*	Sm, Sp	This study
pCSVT22	pRL424 carrying *sepJ-gfpmut2*	Nm	23

**Oligodeoxynucleotide primers[1]**	**Sequence (5′-3′)**
alr2338-BamHI	CGTGGGATCCTTTTCTGTGGTGAGGTGC
gfp-BamHI	AAGCGGATCCTTATTTGTATAGTTCATCCATGCC

[1]The underlined letters indicate a restriction site.
